# Supplementary material for: Differences in regulation mechanisms of glutamine synthetases from methanogenic archaea unveiled by structural investigations
Source: Commun Biol. 2024 Jan 19;7:111. doi: 10.1038/s42003-023-05726-w (PMC10799026; doi:10.1038/s42003-023-05726-w)
Supplement: Supplementary file 2 — Supplementary Information [file 42003_2023_5726_MOESM2_ESM.pdf]

Differences in regulation mechanisms of glutamine synthetases from methanogenic archaea unveiled by structural investigations.

Marie-Caroline Müller, Olivier N. Lemaire, Julia M. Kurth, Cornelia U. Welte, Tristan Wagner*

***Corresponding author. Email:** twagner@mpi-bremen.de

This PDF file includes:

Table S1

Figures S1 to S19

SI references

Other supporting materials for this manuscript include the following:

Excel file of Table S2

Movie “*Mt*GS_conformational_changes.mp4”

Supplementary_data_for_Fig1C_1D_5F.xlsx

Table S1. Oligomeric state prediction by PDBePISA. Predictions were made using the asymmetric units (except for 8OOQ, see below), the two top results being listed in the table. 8OOQ was analyzed as two separate dodecamers, and ligands were removed from 8OOO to facilitate processing.

Structure	PDB code	Monomer / Asymmetric unit	Multimeric state for calculations	ΔG^{int} kcal/mol	ΔG^{diss} kcal/mol
*Mt*GS-apo-TbXo4	8OOL	6	12	-811.42	219.48
			6	-323.76	41.76
*Mt*GS-apo without TbXo4	8OON	6	12	-30.34	149.81
			6	27.22	10.64
*Mt*GS-2OG/Mg^{2+} (A)	8OOQ	2x12	12	-237.25	195.65
			6	-56.28	86.29
*Mt*GS-2OG/Mg^{2+} (B)	8OOQ	2x12	12	-190.32	175.67
			6	-47.76	86.65
*Mt*GS-2OG/Mg^{2+}/ATP	8OOO	12	12	-262.52	189.38
			6	-68.01	79.33
*Ms*GS-apo 1	8OOW	12	12	-348.95	200.48
			6	-103.83	35.98
*Ms*GS-apo 2	8OOX	2	12	-380.81	182.89
			6	-121.07	53.67
*Ms*GS-Mg^{2+}/ATP	8OOZ	12	12	-413.23	219.03
			6	-118.09	44.24

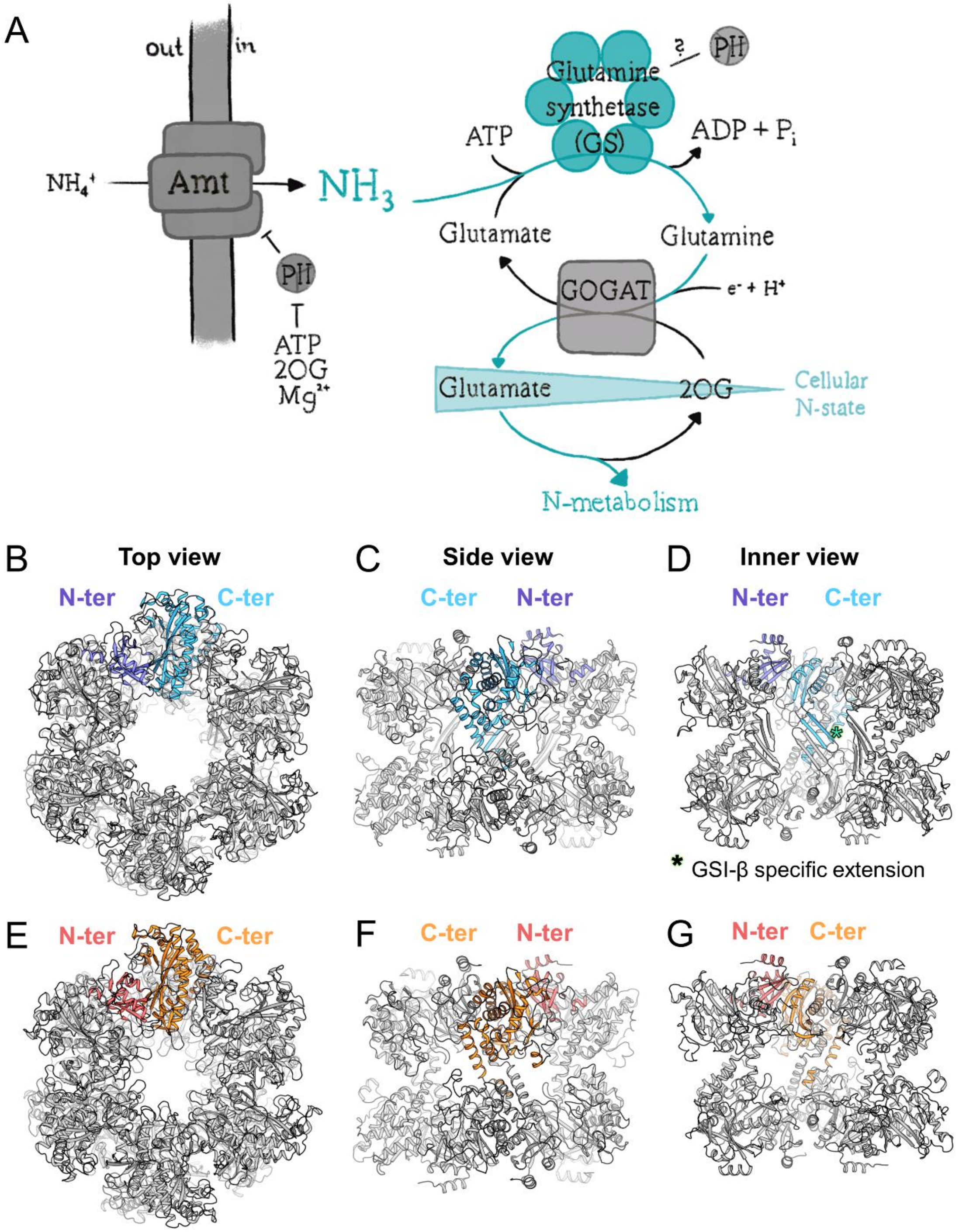

Figure S1: Physiological role of GS-GOGAT in methanogens and structural organization of GSI-α and GSI-β. (A) GS-GOGAT reaction scheme. 2OG concentration increases under nitrogen starvation, while it decreases when cellular nitrogen is available. (B-G) Comparison of homododecameric GSI-β from *Escherichia coli* (B-D, PDB 7W85) and GSI-α from *B. subtilis* (E-G, PDB 4LNN). The proteins are in cartoons with the highlighted N-terminal (dark blue and red) and C-terminal (light blue and orange). (B and E) Top view of the dodecameric GS. (C and F) outer-side view. (D and G) Inner-side view. An asterisk marks the extension characterizing the GSI-β.

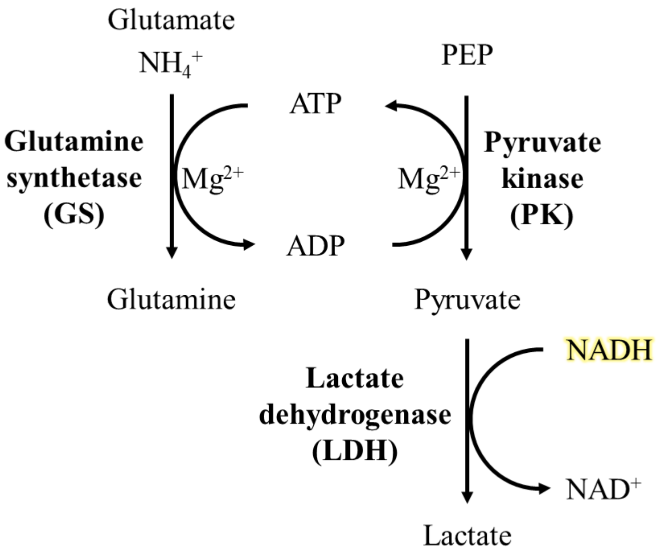

Figure S2: Illustration of the coupled enzyme assay used in this study. The reaction was tracked by following NADH oxidation at 340 nm. Enzyme names are bold. PEP stands for phosphoenolpyruvate. The phosphate liberated by the GS reaction is not shown.

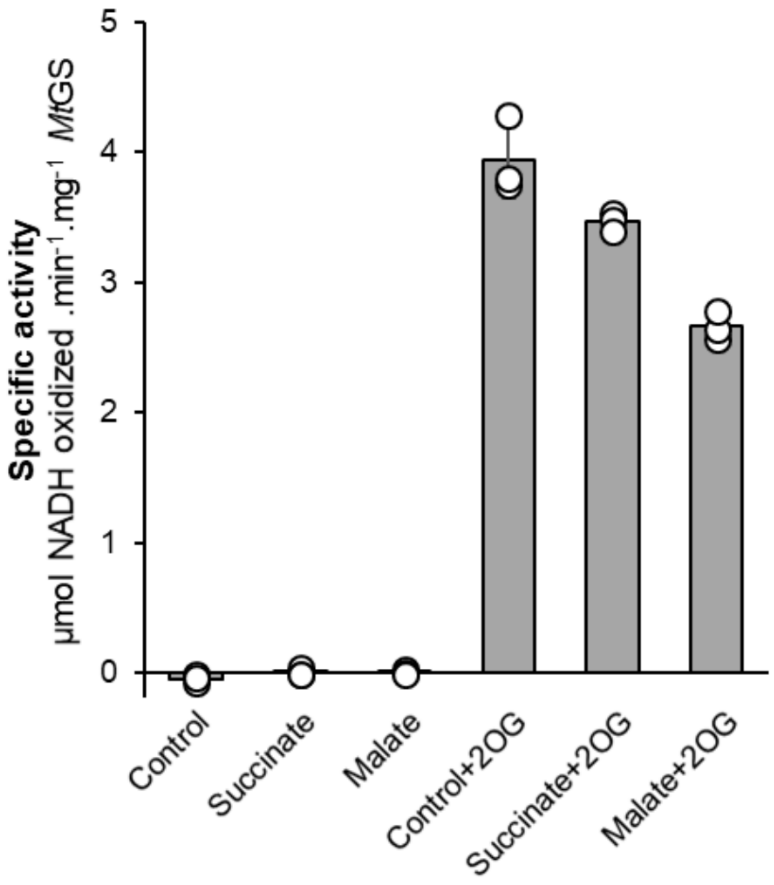

Figure S3: 2OG specificity of *Mt*GS. Specific activity of *Mt*GS after incubation with succinate or malate (15 mM final, first three columns) and after 20 min incubation with succinate and malate (15 mM final) and subsequent 2OG addition (2 mM 2OG, last three columns). Data is represented as mean ± s.d and individual values are shown as white circles (*n*=3).

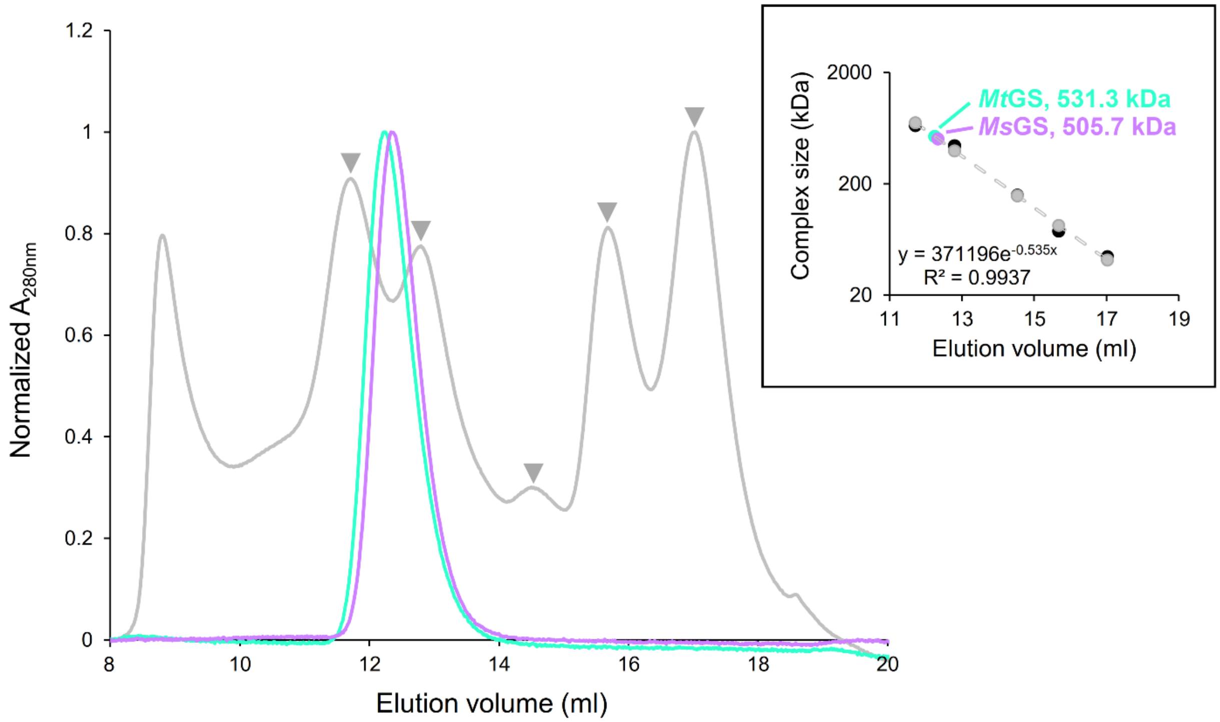

Figure S4. Size exclusion chromatography profile of the purified archaeal GS. Chromatograms of *Mt*GS and *Ms*GS are shown as cyan and purple lines, respectively. The High Molecular Weight (HMW) calibration kit (GE Healthcare) was used as a standard, and its chromatogram is shown as a grey line. Grey arrows indicate the peaks corresponding to the different proteins of the standard used for size estimation: from left to right, thyroglobulin (669 kDa), ferritin (440 kDa), aldolase (158 kDa), conalbumin (75 kDa) and ovalbumin (44 kDa). All chromatograms were normalized for clarity. Inset: determination of the size of the GS complex. The elution volumes of the standard proteins (black dots) were used to establish a fit (dashed grey line) to estimate the molecular weight of GS (cyan and purple dots). Grey dots are experimental calculations of the size of the standard proteins.

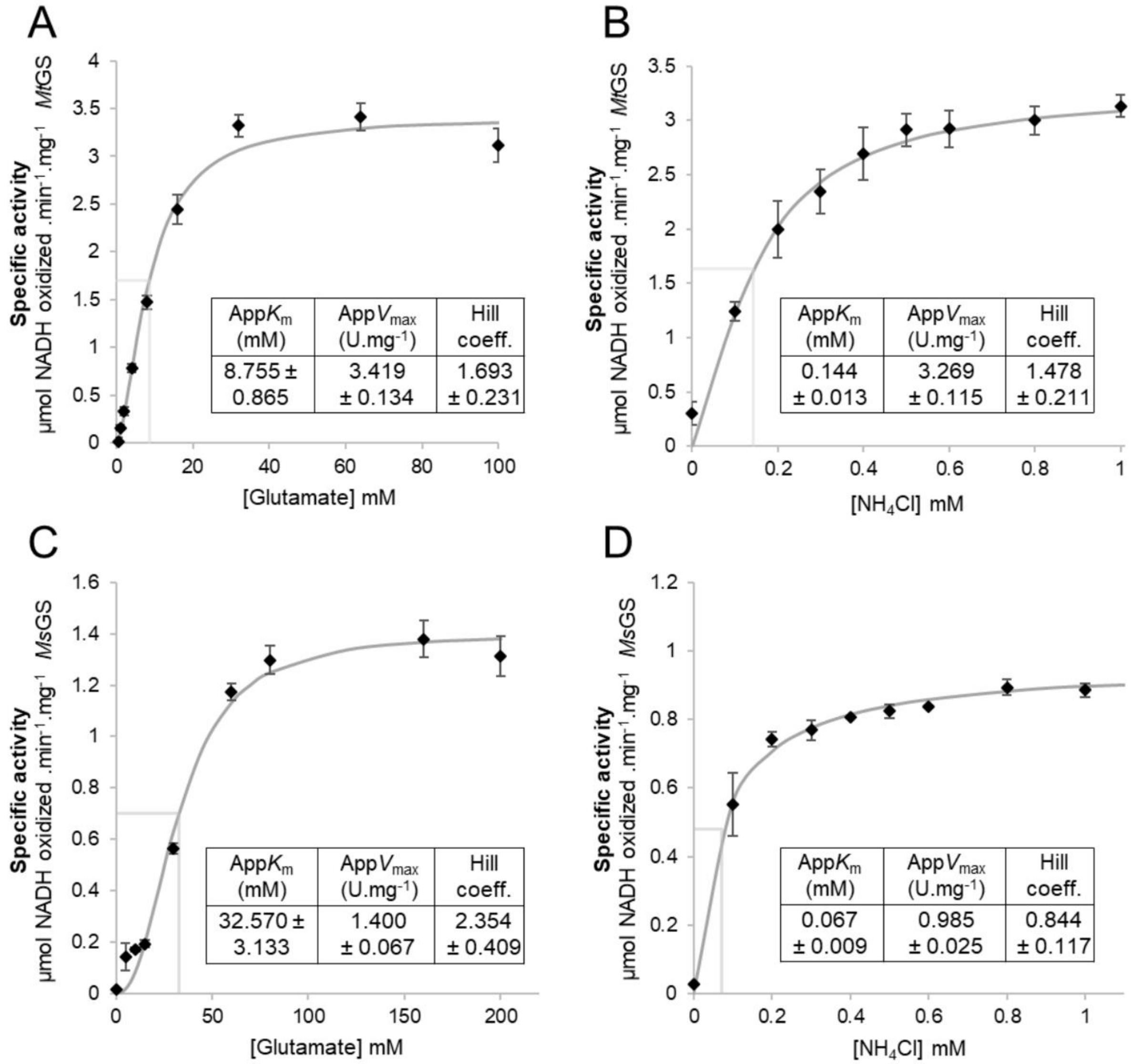

Figure S5: Kinetic parameters of archaeal GS for glutamate and ammonium. Determination of the kinetic parameters of *Mt*GS for glutamate (A) and ammonium (B). Determination of the kinetic parameters of *Ms*GS for glutamate (C) and ammonium (D). The following data points are not shown in the figure for clarity reasons: panel (B) NH_4Cl: 5 mM, specific activity of 3.20 ± 0.15 μmol.min^{-1}.mg^{-1} *Mt*GS and panel (D) NH_4Cl: 2.5 mM, specific activity 0.91 ± 0.02 μmol.min^{-1}.mg^{-1} *Ms*GS and 20 mM, 1.00 ± 0.01 μmol.min^{-1}.mg^{-1} *Ms*GS. Data is represented as mean ± s.d (*n*=3).

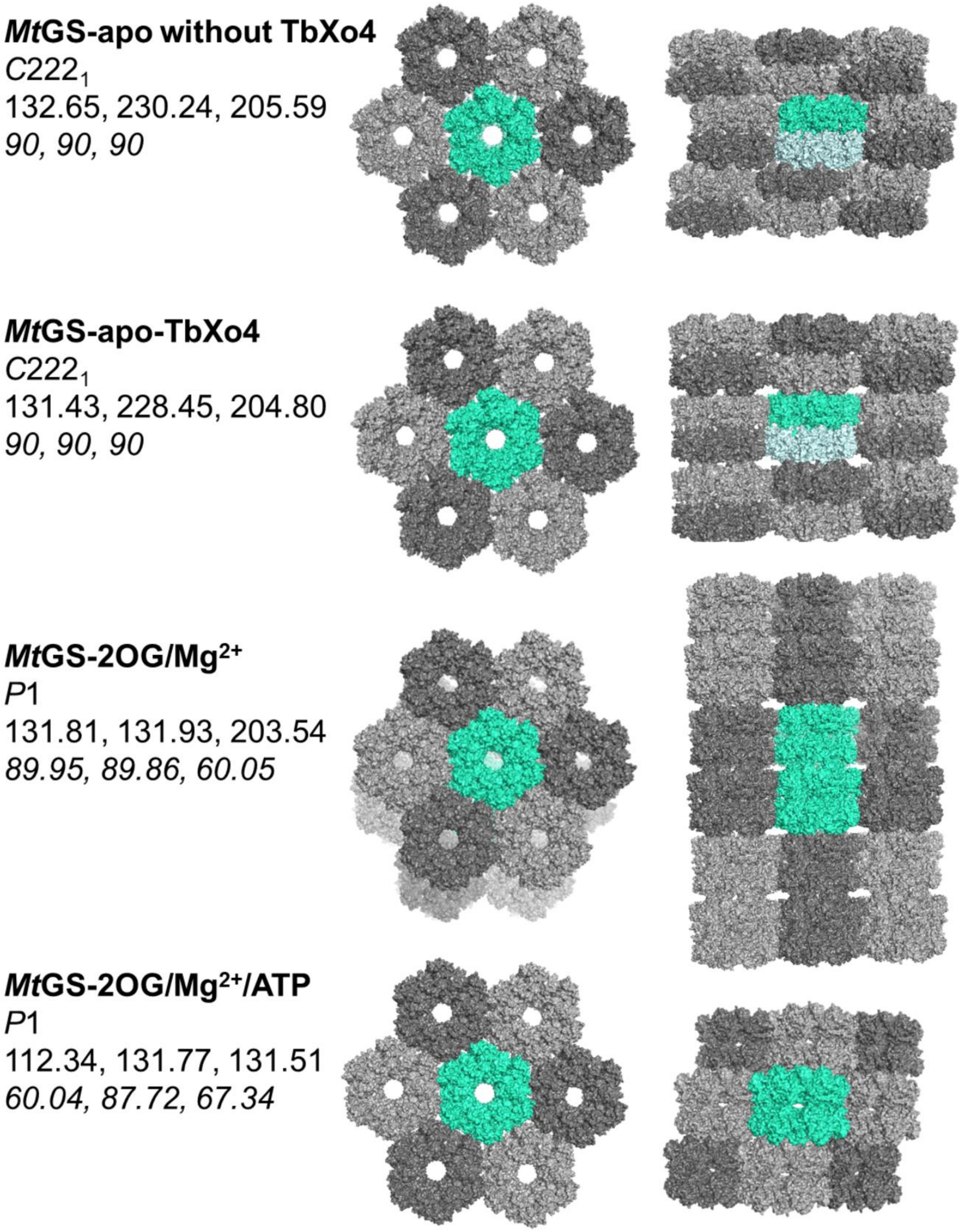

Figure S6: Crystal packing of the *Mt*GS structures. Top and side view of the asymmetric unit in the crystal packing. The asymmetric unit is colored in cyan, symmetry mates are colored in different grey shades, and if the biological dodecamer is generated through a symmetry operator, the additional chains are colored in light blue. The unit cell parameters are given in a (Å), b (Å), c (Å), *α* (°), *β* (°), *γ* (°) format.

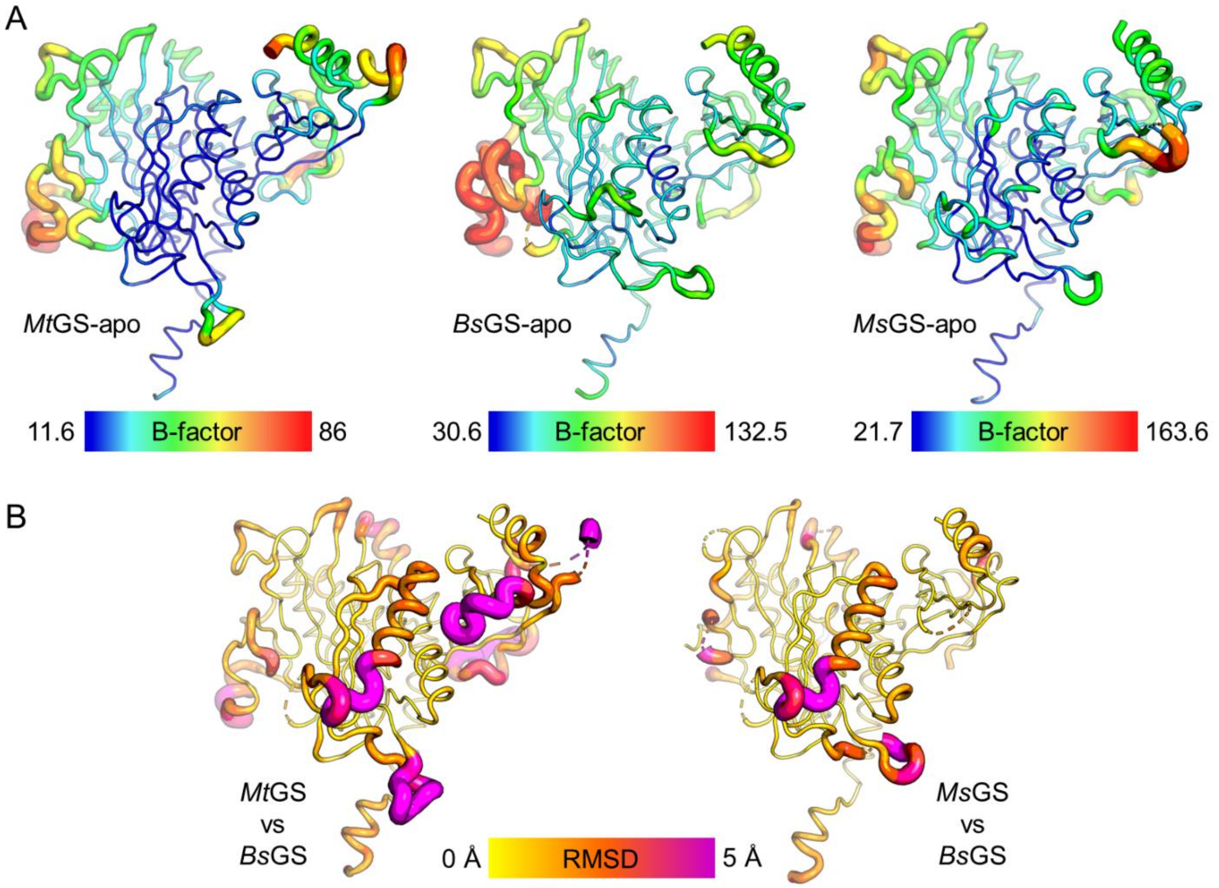

Figure S7: Structural variation between *Mt*GS/*Ms*GS apo and *Bs*GS apo. (A) Cartoon putty of Cα B-factors of the chain with the lowest average B-factor. (B) Root mean square deviation (rmsd) values between *Mt*GS or *Ms*GS with *Bs*GS. Average rmsd values were generated from aligning all chains of *Mt*- or *Ms*GS on chain A of *Bs*GS apo (PDB 4LNN).

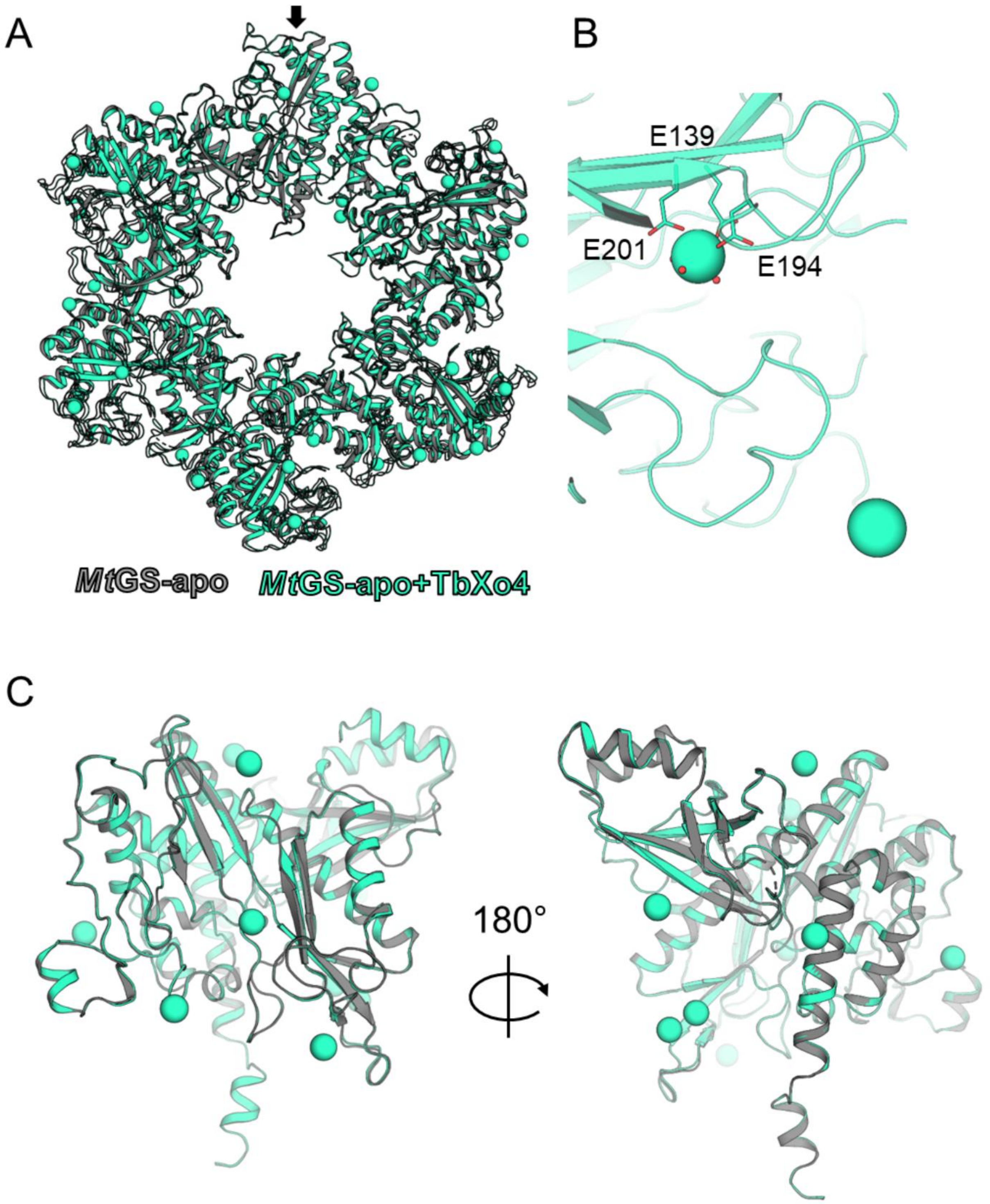

Figure S8: Differences between *Mt*GS apo state with or without TbXo4. (A) Overview of the hexamer from the top. An arrow indicates the subunit used for superposition. (B) Binding of Tb in the active site. (C) Overview of the superposed monomers with the same pose as in Fig. 2c. For A-C, models are represented in cartoon with Tb (cyan) and H_2O as spheres and the coordinating residues as sticks with oxygen atoms in red.

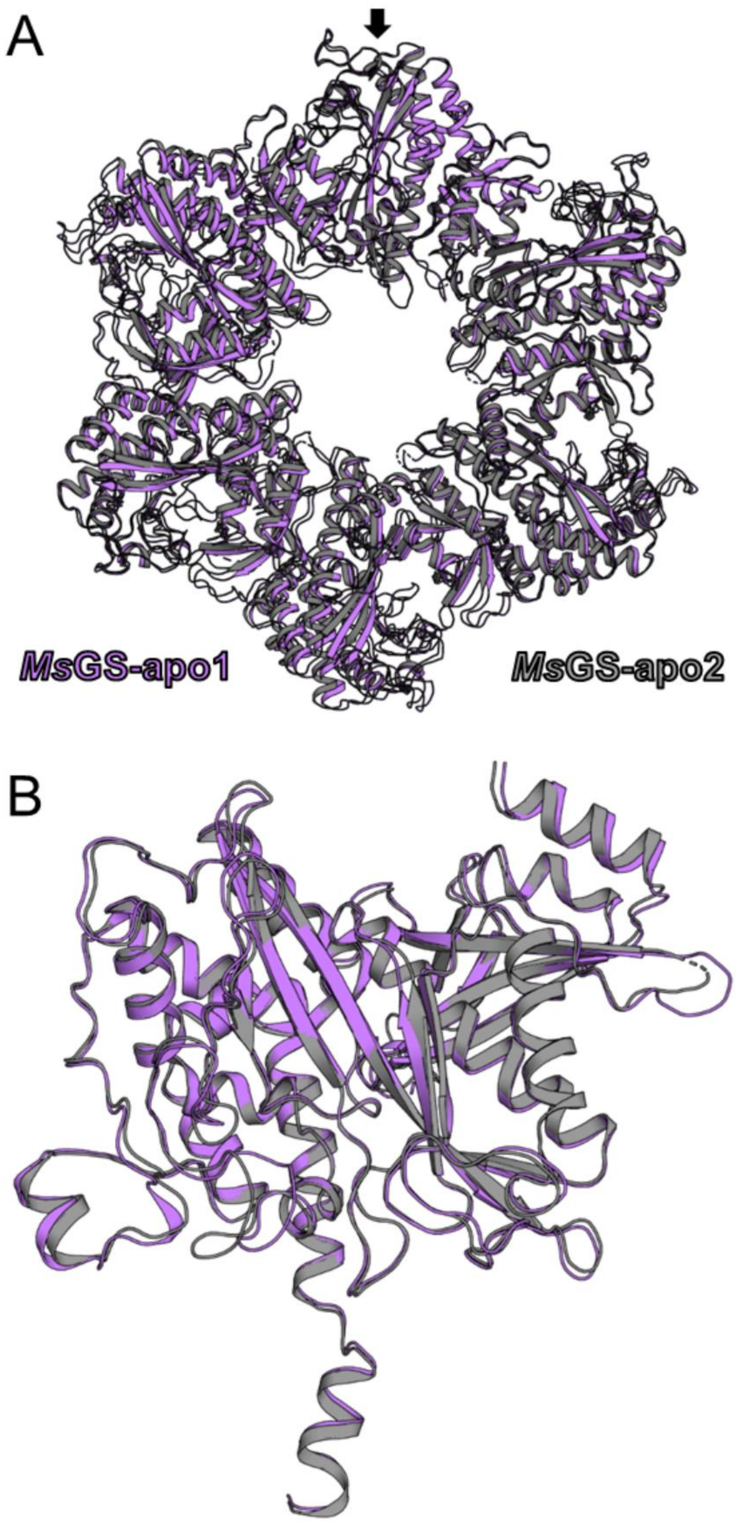

Figure S9: Differences between the two apo forms of *Ms*GS. (A) Superposition of the *Ms*GS apo1 and apo2 hexamers. An arrow indicates the subunit used for superposition. (B) Superposition of the monomers. All models are represented as cartoons with the same pose as in Fig. 2f.

***Ms*GS-apo1**
$P2_1$
130.92, 195.65, 133.44
90, 94.71, 90

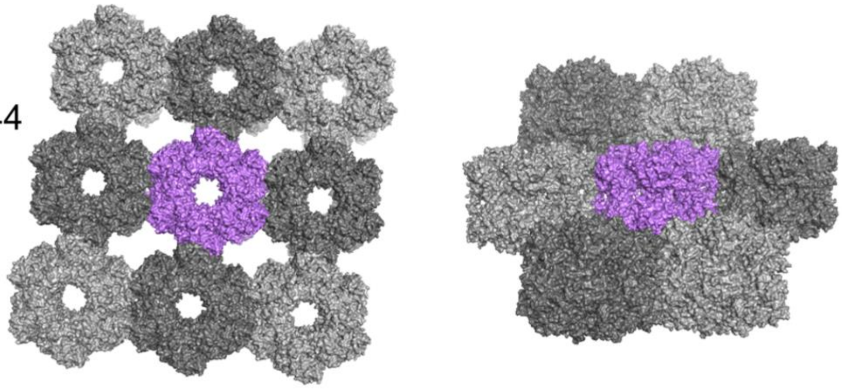

***Ms*GS-apo2**
$P4_332$
226.46, 226.46, 226.46
90, 90, 90

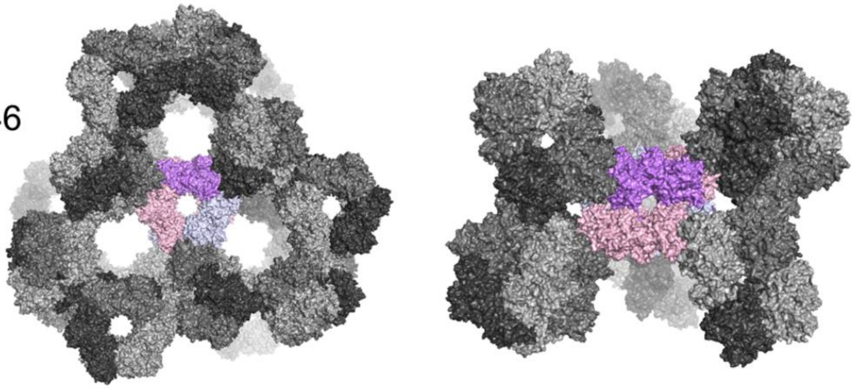

***Ms*GS-Mg^{2+}/ATP**
$P2_1$
131.55, 197.40, 135.17
90, 94.89, 90

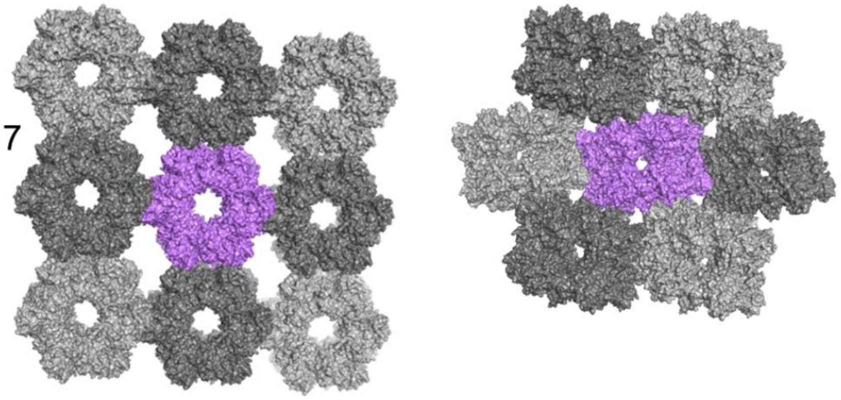

Figure S10: Crystal packing of the obtained *Ms*GS structures. Top and side view of the asymmetric unit in the crystal packing. The asymmetric unit is colored in purple, symmetry mates are colored in different grey shades, and if the biological dodecamer is generated through a symmetry operator, the additional chains are colored in lighter shades of pink. The unit cell parameters are given in a (Å), b (Å), c (Å), *α* (°), *β* (°), *γ* (°) format.

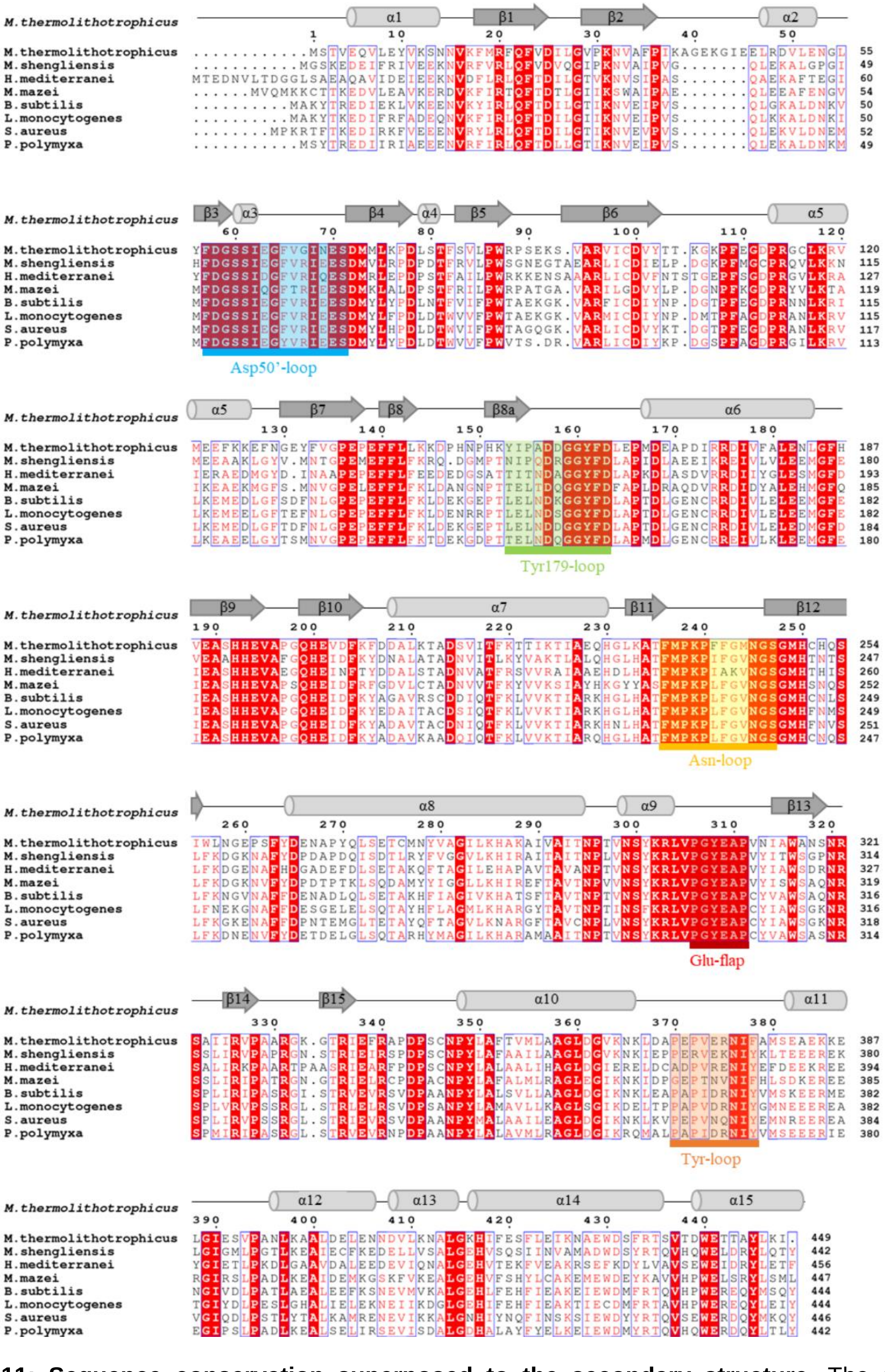

Figure S11: Sequence conservation superposed to the secondary structure. The secondary structure is annotated based on Travis *et al.* 2022 (1). Relevant loops are labeled. WP numbers from top to bottom: WP_018154487.1, WP_042685700.1, CAR67815.1 (*H. mediterranei* GS3), WP_011032914.1 (*M. mazei* GS1), NP_389628.1, EAC9051058.1 (*Listeria monocytogenes*), WP_086038154.1 (*Staphylococcus aureus*), WP_016822091.1 (*Paenibacillus polymyxa*).

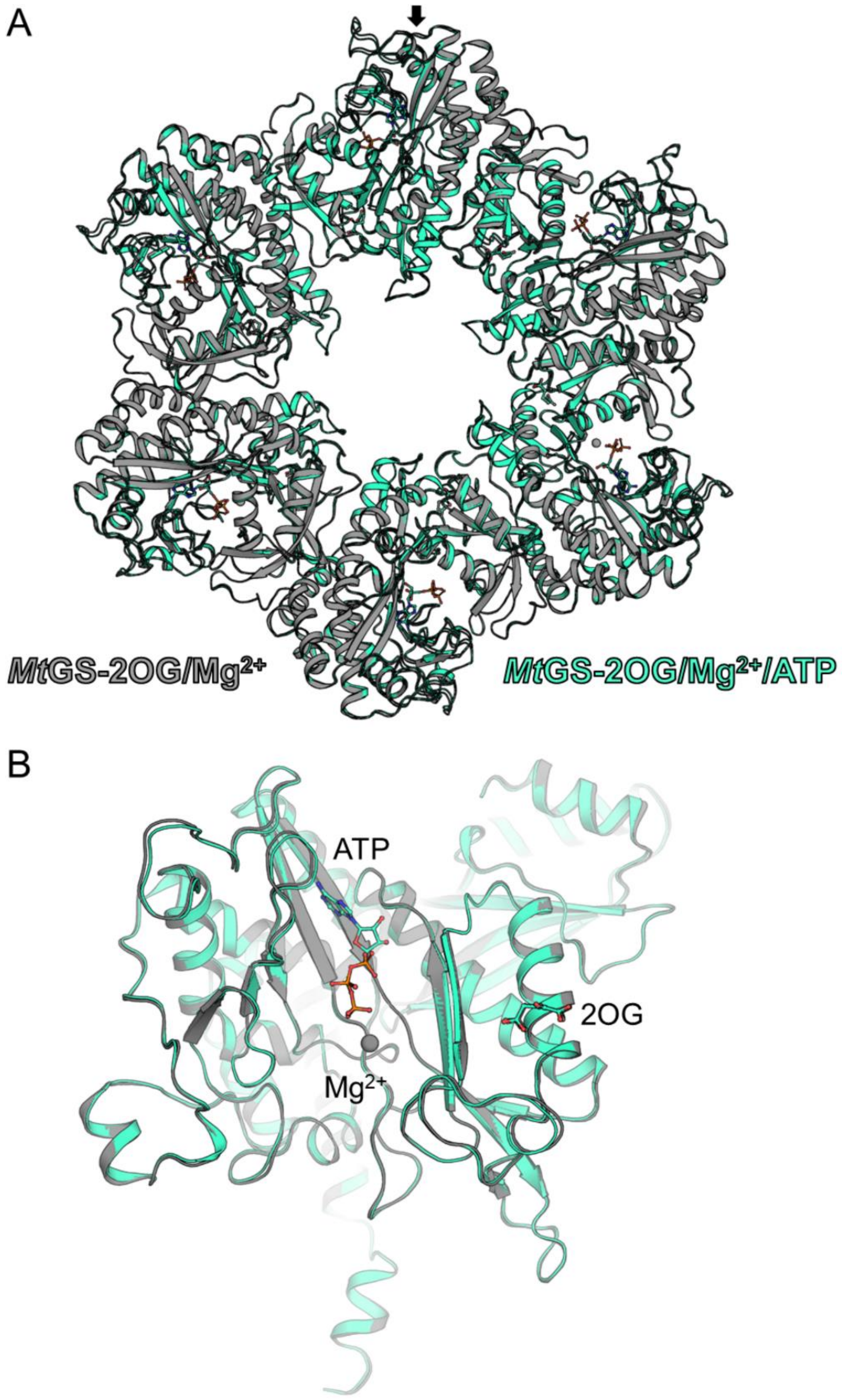

Figure S12: Differences between 2OG/Mg^{2+}-containing and the 2OG/Mg^{2+}/ATP-containing *Mt*GS. (A) Top view of the hexamer aligned on the C-terminal domain. An arrow indicates the subunit used for superposition. (B) Side view of the monomer with the same pose as in Fig. 2c. Models are represented as cartoons with Mg^{2+}, 2OG, and ATP as balls and sticks. Carbon, oxygen, nitrogen, and phosphorus are colored in cyan, red, blue, and orange, respectively.

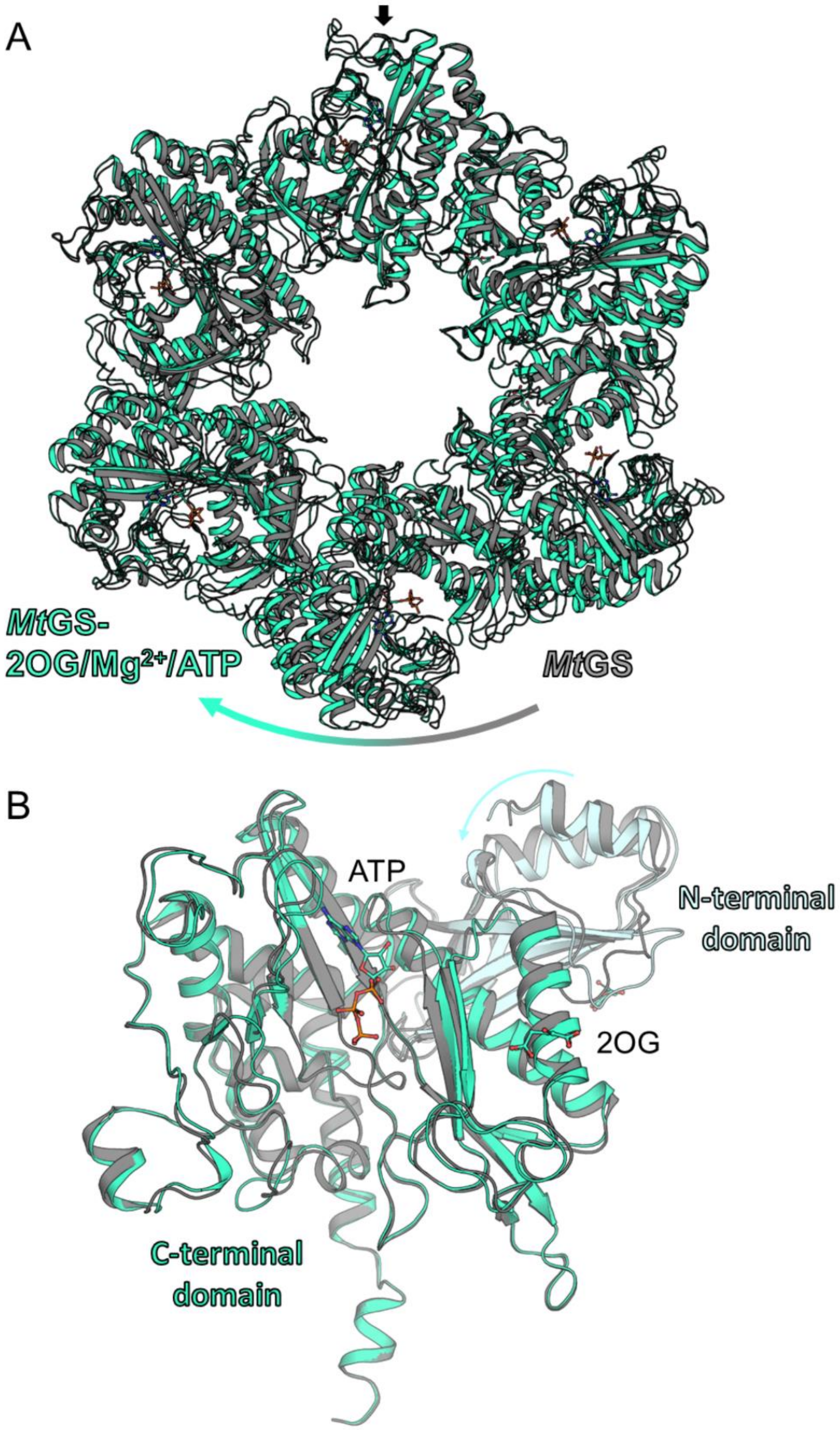

Figure S13: Differences between apo and the 2OG/Mg^{2+}/ATP-containing *Mt*GS. (A) Top view of the superposed *Mt*GS apo and *Mt*GS with 2OG/Mg^{2+}/ATP hexamers. An arrow indicates the subunit used for superposition. (B) Side view of the monomer, aligned on the C-terminal domain with the same pose as in Fig. 2c. Models are represented as cartoons with 2OG, Mg^{2+}, and ATP as balls and sticks. Carbon, oxygen, nitrogen, and phosphorus are colored in cyan, red, blue, and orange, respectively. Structural rearrangements are indicated with arrows.

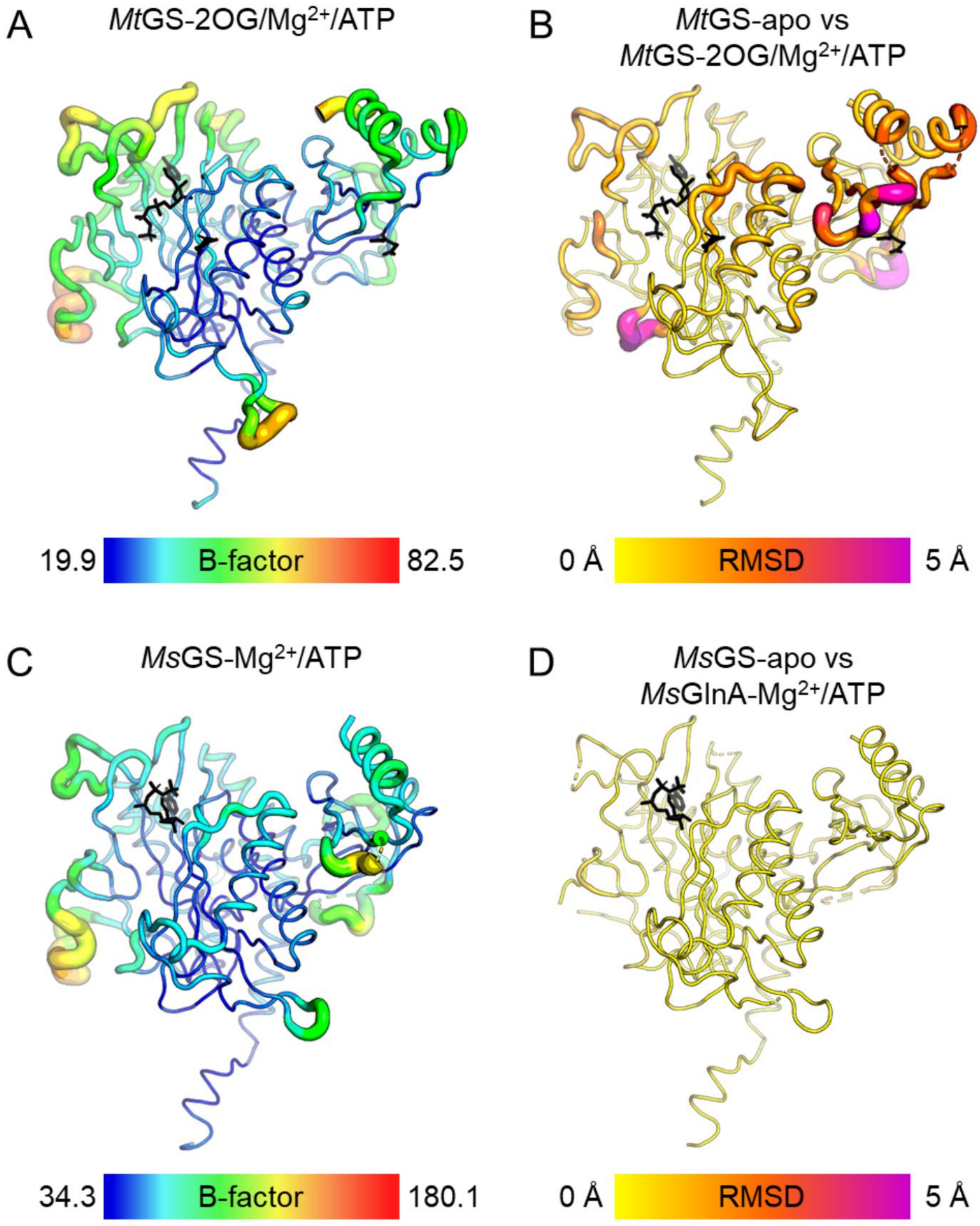

Figure S14: Structural variation between *Mt*GS/*Ms*GS apo and ligand-bound state. (A, C) Cartoon putty of Cα B-factors of the chain with the lowest average B-factor. (B, D) rmsd values with the average of all chains is displayed. *Ms*GS-Mg^{2+}/ATP and *Mt*GS-2OG/Mg^{2+}/ATP *versus* their respective apo state. Ligands are shown as black sticks.

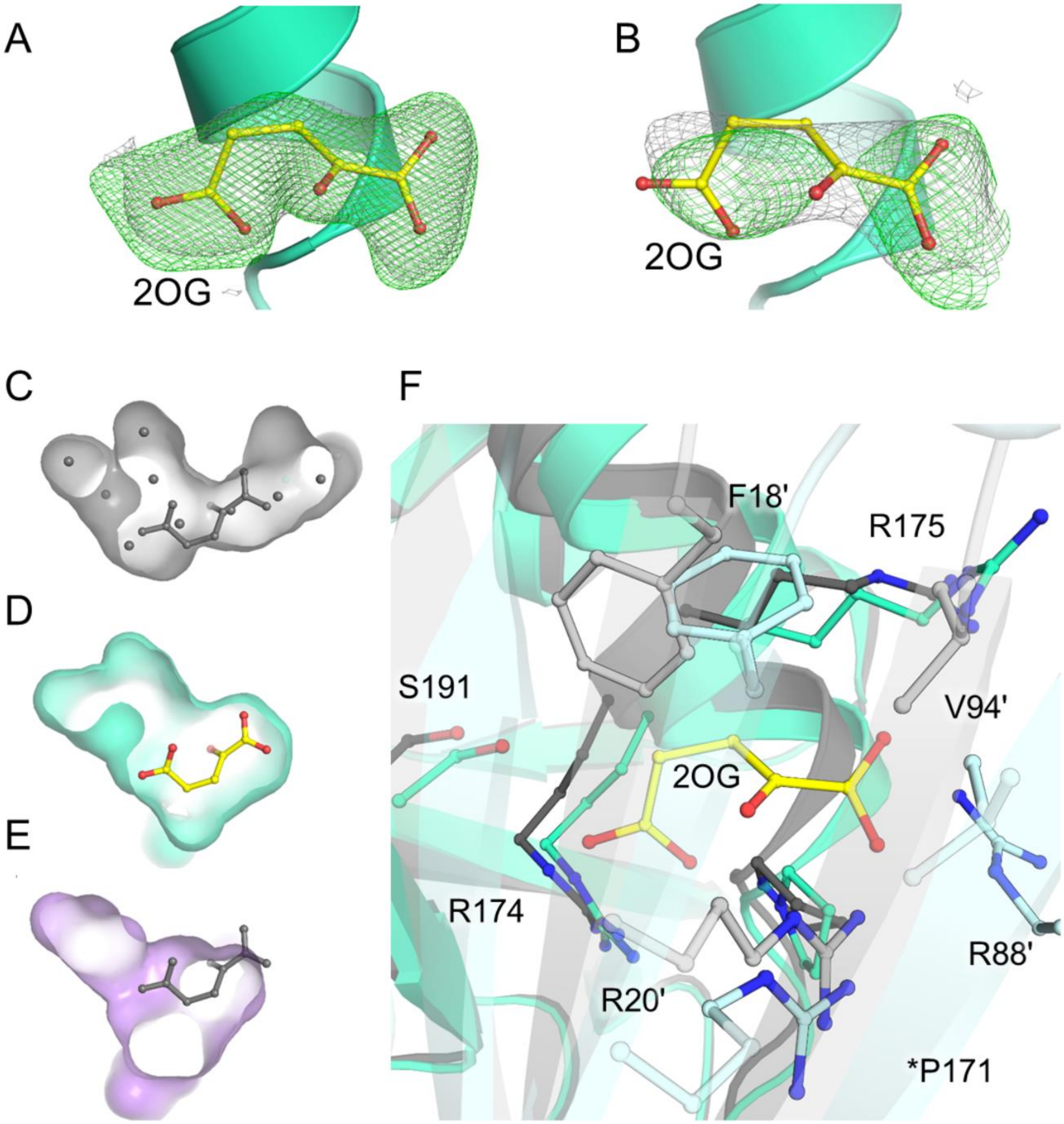

Figure S15: 2OG binding site in *Mt*GS and *Ms*GS. (A and B), Omit maps (2F_o-F_c map contoured at 1 sigma as grey mesh and F_o-F_c map contoured at 3 sigma as green mesh) of 2OG for *Mt*GS-2OG/Mg^{2+}/ATP (A) and *Mt*GS-2OG/Mg^{2+} (B). 2OG are modelled as balls and sticks. (C-E) 2OG pocket surface in *Mt*GS apo (grey), with waters as grey spheres (C), *Mt*GS-2OG/Mg^{2+}/ATP (cyan) (D), and the equivalent position in *Ms*GS-Mg^{2+}/ATP (purple) (E). 2OG was superposed on the C-terminal domain from *Mt*GS-2OG/Mg^{2+}/ATP (grey balls and sticks) for panels C and E. (F) Overlay of *Mt*GS apo (grey) and *Mt*GS-2OG/Mg^{2+}/ATP (cyan). Residues in the close vicinity of 2OG and 2OG itself are shown as balls and sticks. The adjacent subunit is colored in a lighter shade. A, B, and F are represented in cartoons. Oxygen and nitrogen are colored red and blue, respectively. Carbons are colored by chains, and 2OG carbons are yellow. Pro171 is located behind Arg20'.

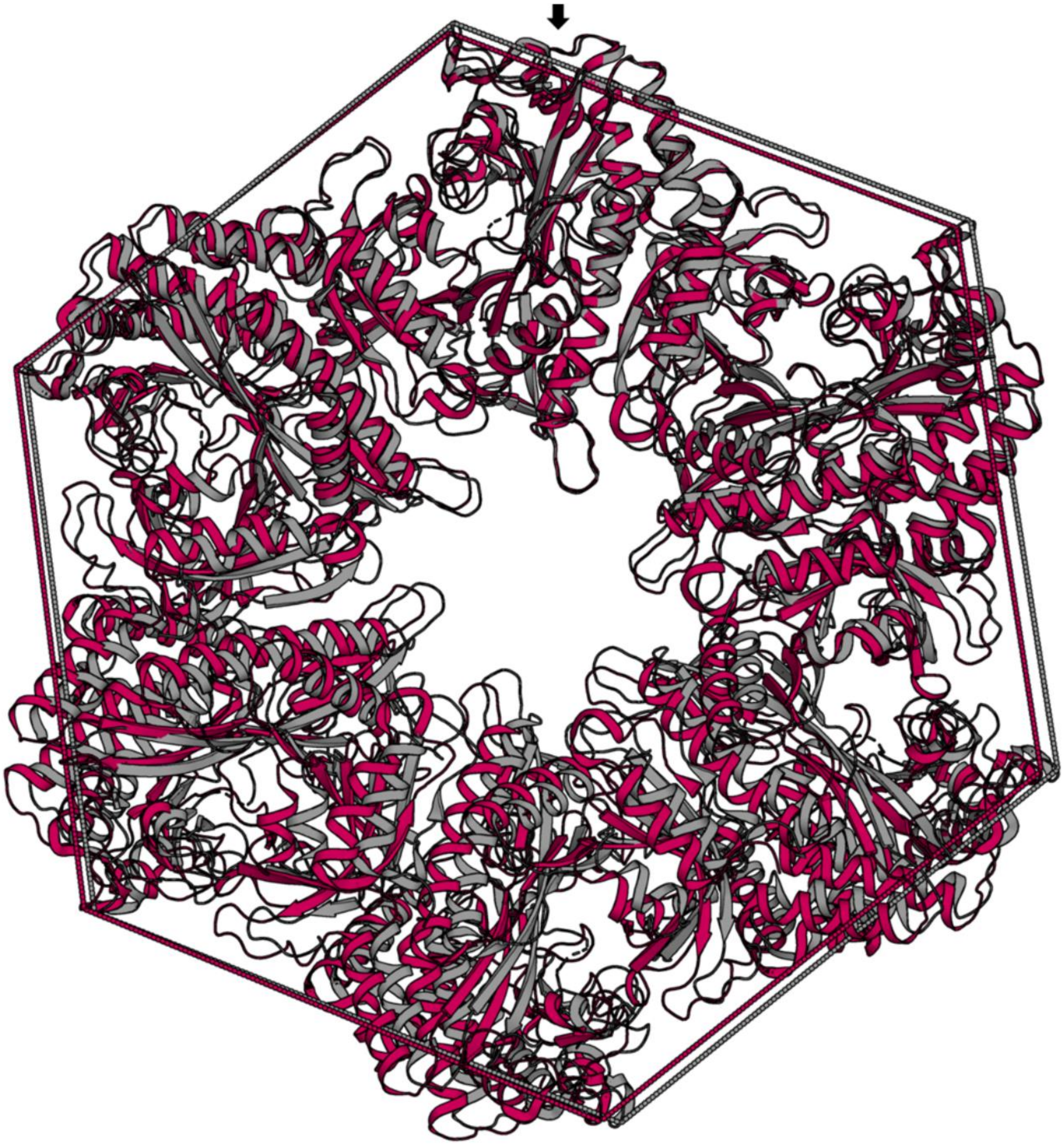

Figure S16: Conformational change in *Bs*GS. Superposition of the *Bs*GS apo (grey, PDB 4LNN) and *Bs*GS containing L-methionine-S-sulfoximine phosphate (SOX, which mimics the reaction intermediate, colored red, PDB 4LNI). Hexamers are represented as cartoons. The superposition was done on one monomer (indicated by an arrow), and a dashed line was drawn on the Cα position of Asn262 to illustrate the overall movements.

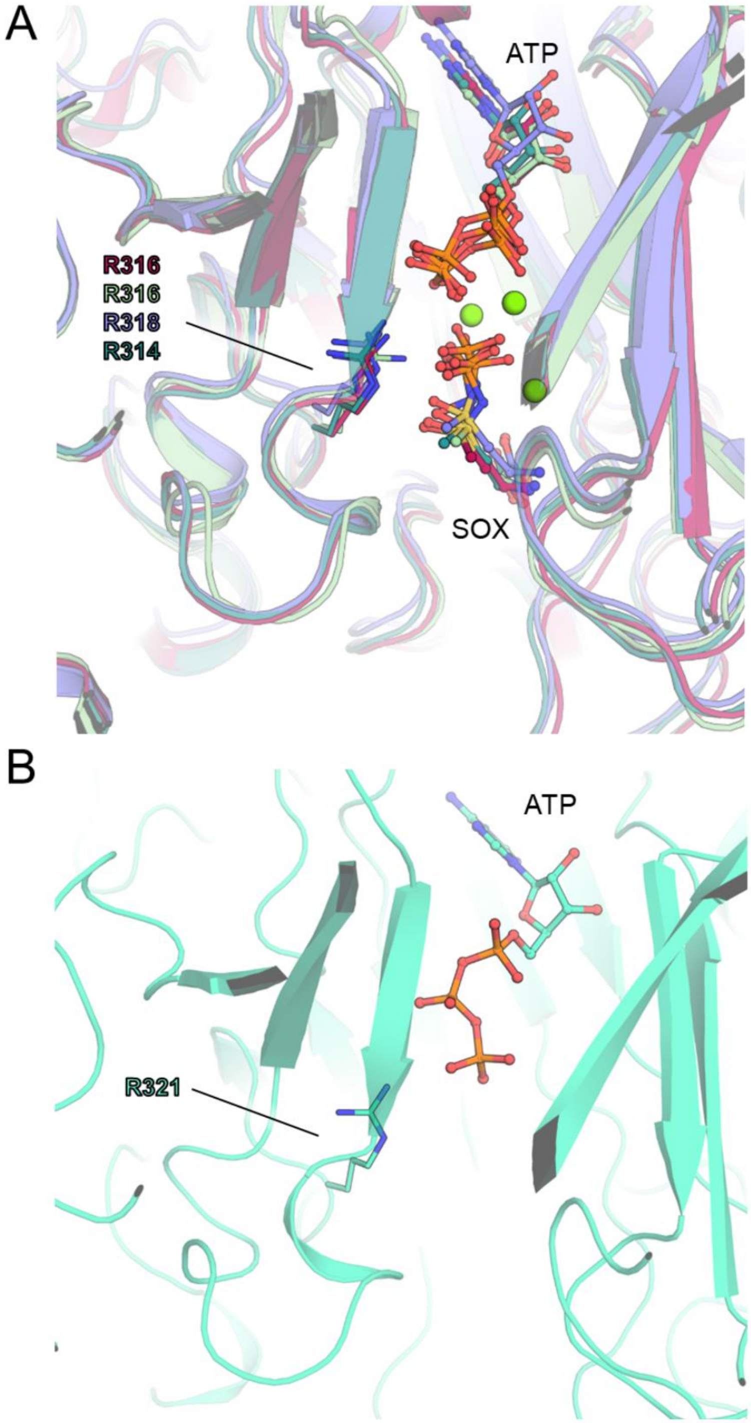

Figure S17: Glutamate binding site view in all structurally characterized bacterial GSI-α and *Mt*GS. (A) *Bs*GS (red, 4LNI), *Staphylococcus aureus* (blue, 7TDV), *Paenibacillus polymyxa* (dark green, 7TDP), *Listeria monocytogenes* (light green, 7TEN), all in the transition state with SOX/Mg^{2+}/ADP bound. (B) *Mt*GS-2OG/Mg^{2+}/ATP. All models are in cartoons with ligands in balls and sticks. Oxygen, nitrogen, sulfur, phosphorus, and magnesium are colored red, blue, yellow, orange and green, respectively. Carbons are accordingly colored to the model. The catalytic arginine is represented as sticks.

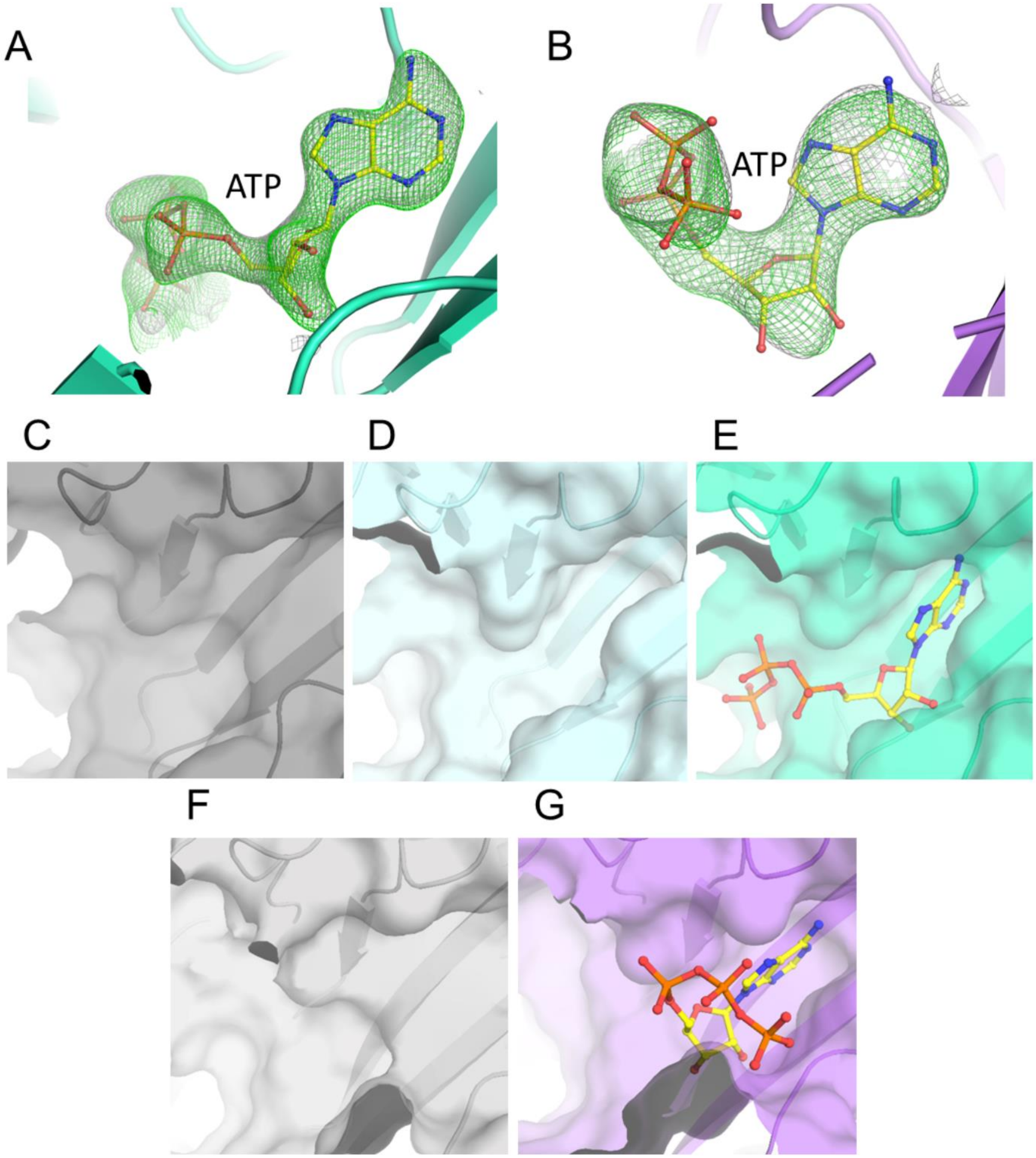

Figure S18: ATP binding pocket in *Mt*GS and *Ms*GS. (A-B) Omit maps ($2F_o$-F_c contoured at 1 sigma, colored in grey mesh and F_o-F_c map contoured at 3 sigma as green mesh) of ATP for *Mt*GS-2OG/Mg^{2+}/ATP (A) and *Ms*GS-Mg^{2+}/ATP (B). (C-E) ATP binding pockets are displayed as transparent surfaces in *Mt*GS apo (C), *Mt*GS-2OG/Mg^{2+} (D), and *Mt*GS-2OG/Mg^{2+}/ATP (E). (F and G) ATP binding pocket in *Ms*GS apo (F) and *Ms*GS-Mg^{2+}/ATP (G). ATP is highlighted as balls and sticks. Carbon, oxygen, nitrogen, and phosphorus are colored yellow, red, blue, and orange, respectively. All models are represented as cartoons.

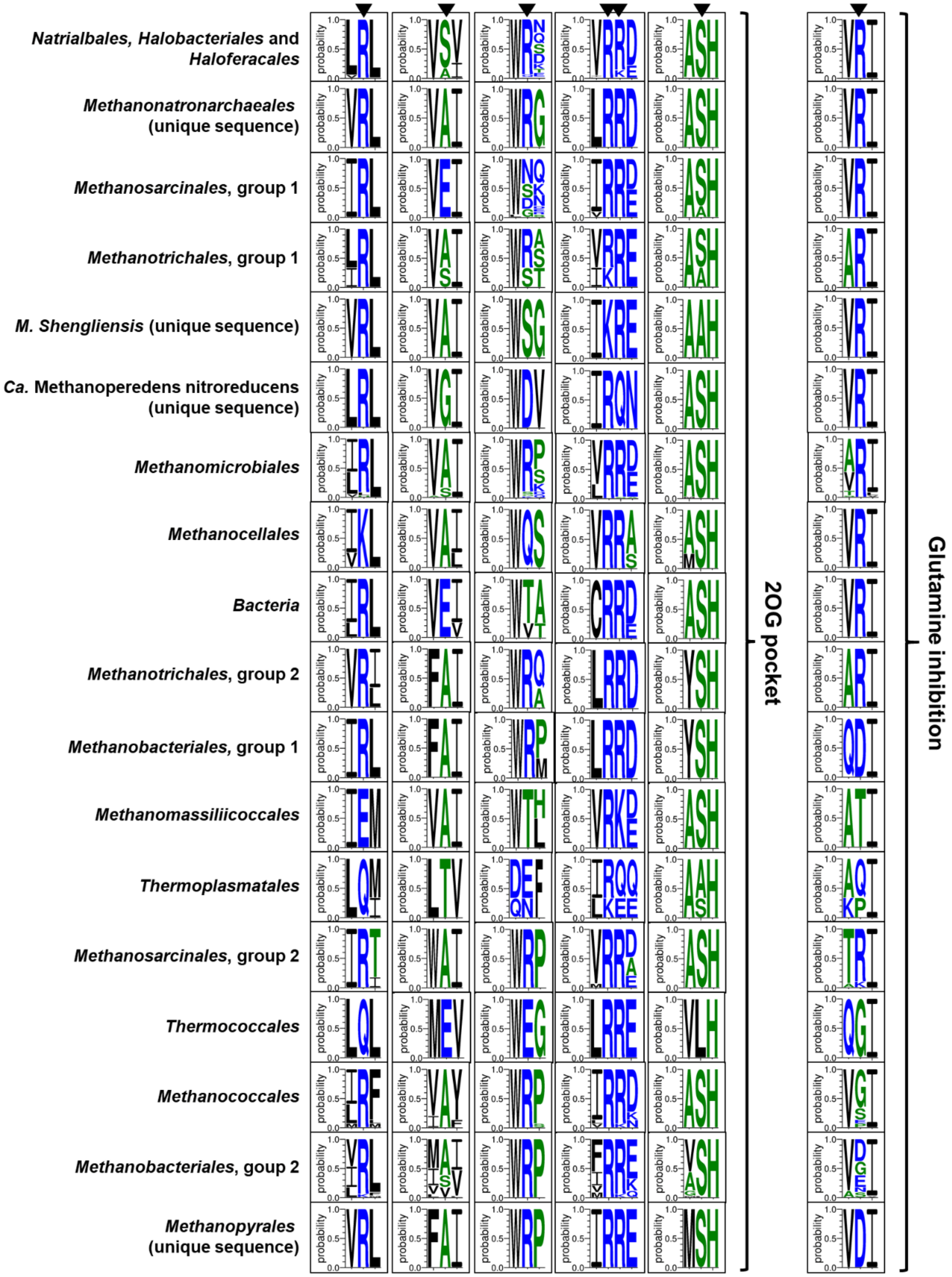

Figure S19: Conservation of residues binding 2OG and glutamine in GSI-α. The sequence conservation at the residues allowing or preventing binding of 2OG and glutamine (according to *Mt*GS, *Ms*GS, and *Bs*GS and shown by black triangles) are illustrated by the Weblogo3 figures constructed on the sequences forming the GS monophyletic groups selected on the tree from Fig. 6.

1. Travis BA, Peck JV, Salinas R, Dopkins B, Lent N, Nguyen VD, et al. Molecular dissection of the glutamine synthetase-GlnR nitrogen regulatory circuitry in Gram-positive bacteria. Nat Commun. 2022;13(1):3793.
