# Supplementary material for: Differences in regulation mechanisms of glutamine synthetases from methanogenic archaea unveiled by structural investigations
Source: Commun Biol. 2024 Jan 19;7:111. doi: 10.1038/s42003-023-05726-w (PMC10799026; doi:10.1038/s42003-023-05726-w)
Supplement: Supplementary file 3 — Description of Additional Supplementary Files [file 42003_2023_5726_MOESM3_ESM.pdf]

Description of Additional Supplementary Files

File name: Supplementary Data 1
Description: Source data for the graphs in Fig 1 and Fig 5.

File name: Supplementary Data 2
Description: The accession numbers of the sequences used for construction of Figure 6 are presented, as the respective organism name and phylum (or the group labelled in the tree). If the accession number refers to several organisms, one was randomly selected.

File name: Supplementary Movie
Description: Movie illustrating the impact of 2OG binding on MtGS.
