# Supplementary material for: Differences in regulation mechanisms of glutamine synthetases from methanogenic archaea unveiled by structural investigations
Source: Commun Biol. 2024 Jan 19;7:111. doi: 10.1038/s42003-023-05726-w (PMC10799026; doi:10.1038/s42003-023-05726-w)
Supplement: Supplementary file 7 — Reporting Summary [file 42003_2023_5726_MOESM7_ESM.pdf]

nature portfolio

Corresponding author(s): Tristan Wagner

Last updated by author(s): Nov 8, 2023

Reporting Summary

Nature Portfolio wishes to improve the reproducibility of the work that we publish. This form provides structure for consistency and transparency in reporting. For further information on Nature Portfolio policies, see our Editorial Policies and the Editorial Policy Checklist.

Statistics

For all statistical analyses, confirm that the following items are present in the figure legend, table legend, main text, or Methods section.

n/a	Confirmed	
☐	☒	The exact sample size (*n*) for each experimental group/condition, given as a discrete number and unit of measurement
☐	☒	A statement on whether measurements were taken from distinct samples or whether the same sample was measured repeatedly
☒	☐	The statistical test(s) used AND whether they are one- or two-sided<br>*Only common tests should be described solely by name; describe more complex techniques in the Methods section.*
☒	☐	A description of all covariates tested
☒	☐	A description of any assumptions or corrections, such as tests of normality and adjustment for multiple comparisons
☐	☒	A full description of the statistical parameters including central tendency (e.g. means) or other basic estimates (e.g. regression coefficient) AND variation (e.g. standard deviation) or associated estimates of uncertainty (e.g. confidence intervals)
☒	☐	For null hypothesis testing, the test statistic (e.g. *F*, *t*, *r*) with confidence intervals, effect sizes, degrees of freedom and *P* value noted<br>*Give P values as exact values whenever suitable.*
☒	☐	For Bayesian analysis, information on the choice of priors and Markov chain Monte Carlo settings
☒	☐	For hierarchical and complex designs, identification of the appropriate level for tests and full reporting of outcomes
☒	☐	Estimates of effect sizes (e.g. Cohen's *d*, Pearson's *r*), indicating how they were calculated

Our web collection on statistics for biologists contains articles on many of the points above.

Software and code

Policy information about availability of computer code

Data collection	X-ray data collection was performed at the Swiss Light Source (SLS), beamline PXIII (X06DA). Activity data were determined with a SPECTROstar Nano (BMG Labtech). Absorbances of cultures and Bradford assays were measured with a UVmini-1240 UV-vis spectrophotometer (Shimadzu). pH values were determined with a pH-Meter 766 Calimatric (Knick). Purifications were carried out on an Äkta pure (Cytiva) and monitored with UNICORN 7.3. (General electric company).
Data analysis	Enzyme activities were calculated with Excel 2016 (Microsoft Office). Structure data processing was performed with autoPROC (Global phasing), STARANISO (Global phasing), XDS (CCP4) and Scala (CCP4). Molecular replacement was performed with MOLREP (CCP4) and PHASER (PHENIX). Refinement was performed with Phenix refine and BUSTER (Global phasing) in combination with fast, automatic visual model building in COOT (Emsley, P.; Lohkamp, B.; Scott, W. G.; Cowtan, K., Features and development of Coot. Acta Crystallographica Section D-Biological Crystallography 2010, 66, 486-501). Models were validated by using Molprobity (http://molprobity.biochem.duke.edu/). All structure visualization was performed with PyMol open source (Schrödinger). RMSD values were calculated with SUPERPOSE (CCP4). The movie was generated with UCSF Chimera (University of California). Sequence alignments were generated with Clustal Omega (https://www.ebi.ac.uk/Tools/msa/clustalo/) and superposed to the secondary structure with the Espript 3.0 server (https://espript.ibcp.fr). Archaeal GS sequences were retrieved via BLAST P (https://blast.ncbi.nlm.nih.gov/Blast.cgi?PAGE=Proteins), alignments were generated with MUSCLE (https://www.ebi.ac.uk/Tools/msa/muscle/), the phylogenic tree was constructed by MEGA X (https://www.megasoftware.net/) and visualized with iTol v6 (https://itol.embl.de/). GS sequences within groups were aligned with Clustal Omega (https://www.ebi.ac.uk/Tools/msa/clustalo/), and residue conservation images were constructed using Weblogo 3 (https://weblogo.berkeley.edu/logo.cgi).

For manuscripts utilizing custom algorithms or software that are central to the research but not yet described in published literature, software must be made available to editors and reviewers. We strongly encourage code deposition in a community repository (e.g. GitHub). See the Nature Portfolio guidelines for submitting code & software for further information.

Data

Policy information about availability of data

All manuscripts must include a data availability statement. This statement should provide the following information, where applicable:

- Accession codes, unique identifiers, or web links for publicly available datasets
- A description of any restrictions on data availability
- For clinical datasets or third party data, please ensure that the statement adheres to our policy

All protein models were deposited with the Worldwide Protein Data Bank (wwPDB) under the accession numbers 8OOL, 8OON, 8OOO, 8OOQ, 8OOW, 8OOX and 8OOZ.

Research involving human participants, their data, or biological material

Policy information about studies with human participants or human data. See also policy information about sex, gender (identity/presentation), and sexual orientation and race, ethnicity and racism.

Reporting on sex and gender	Not applicable
Reporting on race, ethnicity, or other socially relevant groupings	Not applicable
Population characteristics	Not applicable
Recruitment	Not applicable
Ethics oversight	Not applicable

Note that full information on the approval of the study protocol must also be provided in the manuscript.

Field-specific reporting

Please select the one below that is the best fit for your research. If you are not sure, read the appropriate sections before making your selection.

☒ Life sciences ☐ Behavioural & social sciences ☐ Ecological, evolutionary & environmental sciences

For a reference copy of the document with all sections, see nature.com/documents/nr-reporting-summary-flat.pdf

Life sciences study design

All studies must disclose on these points even when the disclosure is negative.

Sample size	Purifications were carried out with adapted protocols sequentially. Activities were determined on single samples in technical triplicates.
Data exclusions	No data was excluded.
Replication	Replicates are stated in the methods.
Randomization	Randomization was applied for the generation of the Rfree set for structure refinement.
Blinding	Not applicable

Reporting for specific materials, systems and methods

We require information from authors about some types of materials, experimental systems and methods used in many studies. Here, indicate whether each material, system or method listed is relevant to your study. If you are not sure if a list item applies to your research, read the appropriate section before selecting a response.

Materials & experimental systems

n/a	Involved in the study
☒	☐ Antibodies
☒	☐ Eukaryotic cell lines
☒	☐ Palaeontology and archaeology
☒	☐ Animals and other organisms
☒	☐ Clinical data
☒	☐ Dual use research of concern
☒	☐ Plants

Methods

n/a	Involved in the study
☒	☐ ChIP-seq
☒	☐ Flow cytometry
☒	☐ MRI-based neuroimaging
